# Mechanistic assessment of cadmium toxicity in association with the functions of estrogen receptors in the Langerhans islets

**DOI:** 10.22038/ijbms.2019.33939.8076

**Published:** 2019-04

**Authors:** Perham Mohammadi, Mahban Rahimifard, Maryam Baeeri, Mohammad Abdollahi, Sara Mostafalou

**Affiliations:** 1 Department of Pharmacology and Toxicology, School of Pharmacy, Ardabil University of Medical Sciences, Ardabil, Iran; 2 Toxicology and Diseases Group, The Institute of Pharmaceutical Sciences (TIPS), Tehran University of Medical Sciences, Tehran, Iran; 3 Department of Toxicology and Pharmacology, Faculty of Pharmacy, Tehran University of Medical Sciences, Tehran, Iran

**Keywords:** Cadmium, Diabetes mellitus, Estradiol, Estrogen receptors, Insulin, Islets of Langerhans Pancreas

## Abstract

**Objective(s):**

Diabetes is a metabolic disease with an increasing prevalence for which finding new and efficient therapeutic approaches has always been a challenge. Preserving integrity and functionality of pancreatic β-cells as the only source of insulin in the body is such a case. To achieve this goal different cellular targets have been proposed among which pancreatic estrogen receptors have gotten much attention. In this work, we evaluated the integrity and function of islets of Langerhans under the influence of factors known to intervene with estrogen receptors. Cadmium, a toxic heavy metal, has been recently shown to interact with estrogen receptors but its toxicity in the pancreatic islets regarding this mechanism remains unclear.

**Materials and Methods:**

Isolated islets of Langerhans from the pancreas of rats were grouped and treated with cadmium chloride and also cadmium chloride plus β-estradiol. After 24 hr incubation, parameters of cellular viability, oxidative stress, apoptosis, and insulin secretion were measured***. ***

**Results:**

The results indicated that cadmium reduced viability of the islets along with an increase in the formation of reactive oxygen species and apoptosis markers, and β-estradiol, in turn, was able to alleviate these disturbances to some extent, implicating the protective role of β-estradiol against pancreatic toxicity of cadmium.

**Conclusion:**

It can be concluded that modification of estrogen receptors in the endocrine pancreas and especially β-cells may be a promising target to find a new therapeutic strategy for diabetes and even uncovering mechanisms of environmental toxicants that have been known as risk factors of diabetes.

## Introduction

As a chronic and metabolic disease, diabetes has risen dramatic in prevalence. The first Global report of WHO on diabetes showed that the prevalence of diabetes has almost quadrupled since 1980, and 1.5 million annual deaths are directly attributed to it (WHO). In parallel with this rise, local and global investigations are increasing to explore new risk factors, pathologic mechanisms, and therapeutic strategies. The main risk factors for diabetes include high-calorie diet, physical inactivity, and genetic factors. According to a huge body of evidence, exposure to environmental toxicants like pesticides and persistent organic pollutants are considered risk factors of endocrine and metabolic diseases such as diabetes (1-5). Exposure to heavy metals have also been known as one of the main environmental problems for human health, and recently lots of evidence have been presented regarding their association with diabetes (6). 

Cadmium is one of the main heavy metals having different industrial applications, and human exposure to its toxic levels can result in renal and skeletal disorders. Recently, cellular and molecular investigations on cadmium and some other heavy metals have revealed that these compounds can disrupt endocrine homeostasis through interacting with estrogen receptors. Thus, the phrase “metallo-hormone” is created and used to specify those metals interacting with receptors and other elements of the endocrine system (7). 

On the other hand, it has been made clear that some types of estrogen receptors are available in the pancreatic β-cells whose vital function is synthesis and secretion of insulin as the chief hormone controlling glucose metabolism in the body. Although no definite function has been specified for estrogen receptors in the pancreas, protective roles of these receptors for viability and integrity of β-cells as well as controlling biosynthesis and secretion of insulin have been proposed and defined by some studies (8). In this way, modification of estrogen receptors in the pancreatic β-cells has been suggested as a new approach for management of diabetes, but diverse expression of these receptors in different organs and their multifarious functions in the endocrine system have created a need for more studies on the exact pharmacological and toxicological positions of estrogen receptors in diabetes. 

Furthermore, cadmium has been shown to be accumulated in the pancreatic β-cells at environmentally relevant concentrations and disrupt their insulin-secreting function without affecting cellular viability (9). According to the evidence implicating the interaction of cadmium with estrogen receptors on the one hand and the role of these receptors in preserving the integrity and biological function of pancreatic β-cells on the other, there is a need to study cadmium-induced pancreatic toxicity through interacting with the functions of estrogenic receptors in the pancreatic β-cells. 

The objective of this work was to study glucotoxicity of cadmium as a proposed metallo-hormone with regard to the function of estrogen receptors in the pancreas. For this purpose, isolated islets of Langerhans from the pancreas of rats have been evaluated from the aspects of cellular viability, oxidative stress, apoptosis, and insulin secretion under the effect of cadmium both alone and along with β-estradiol as an endogenous agonist of estrogen receptors. 

## Materials and Methods

***Chemicals***

Chemicals including cadmium chloride, β-estradiol, 3-(4,5-dimethylthiazol-2-yl)-2,5-diphenyltetrazolium bromide (MTT), RPMI 1640 media, Pen-Strep, glucose, collagenase, HEPES sodium salt, bovine serum albumin (BSA), ethidium bromide (EB) and 2′,7′-dichlorodihydrofluorescein diacetate (DCFH-DA) were procured from the Sigma-Aldrich Company (GmbH Munich, Germany). The ApoFlowEx® FITC Kit was obtained from antibodies-online while a rat-specific insulin ELISA kit was acquired from Mercodia (Sweden). 

**Figure 1:**
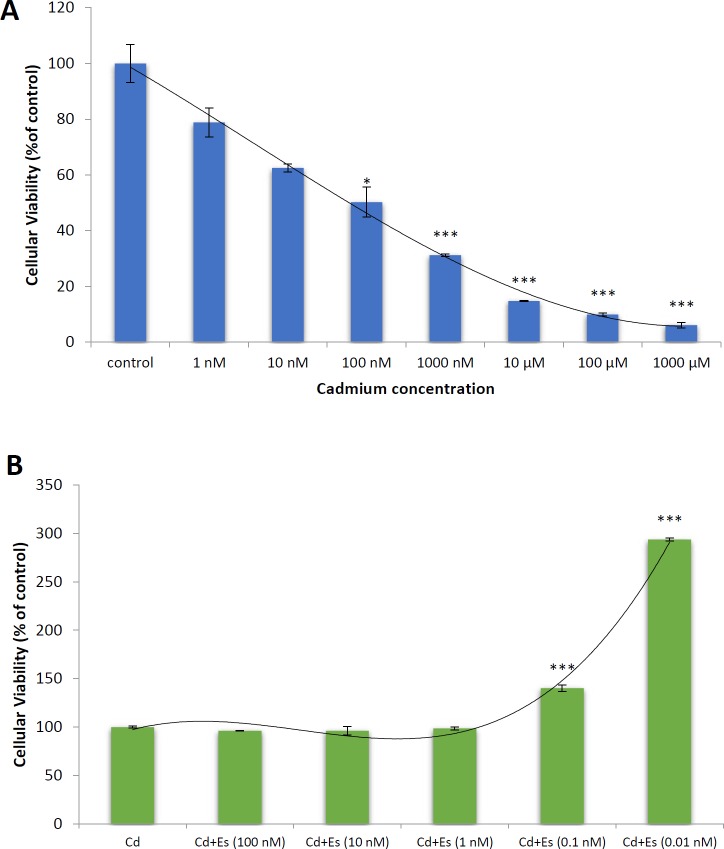
Cellular viability of isolated islets of Langerhans from the rat pancreas after exposure to cadmium chloride (A) and cadmium chloride (100 nM) plus β-estradiol (B). Values are expressed as mean ± SEM. * significantly different from control at *P*-value < 0.05, ***significantly different from control at *P*-value < 0.001. Cd: cadmium, Es: β-estradiol

**Figure 2:**
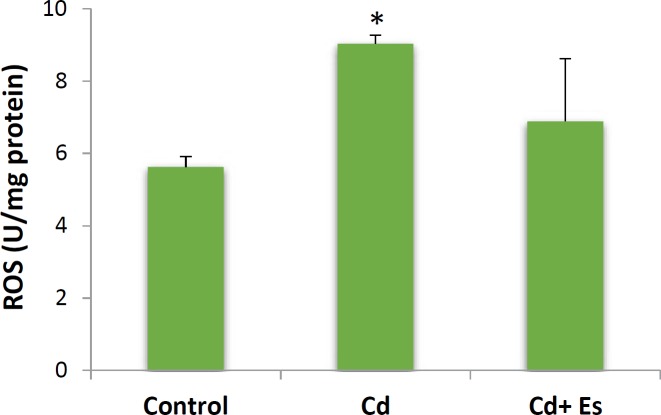
ROS formation in isolated islets of Langerhans from the rat pancreas after exposure to cadmium chloride and cadmium chloride plus β-estradiol. Values are expressed as mean±SEM. * significantly different from control at *P*-value < 0.05. Cd: cadmium, Es: β-estradiol, ROS: reactive oxygen species

**Figure 3:**
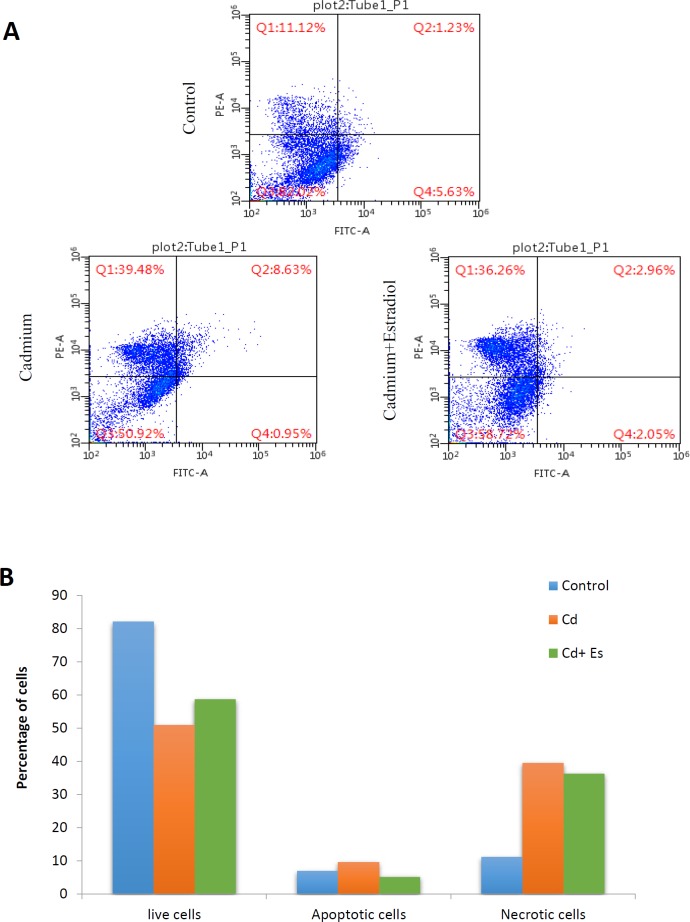
Flow cytometry assessment of live, early apoptotic, late apoptotic, and necrotic cells of rat islets of Langerhans exposed to cadmium chloride and β-estradiol. Lower left field (FITC− and PI−) shows live cells, lower right field (FITC+ and PI−) indicates early apoptotic cells, upper right field (FITC+ and PI+) represents late apoptotic cells, and upper left field (FITC− and PI+) expresses necrotic cells (A). Percentage of cells in the stages of live, apoptosis, and necrosis (B). Cd: cadmium, Es: β-estradiol

**Figure 4:**
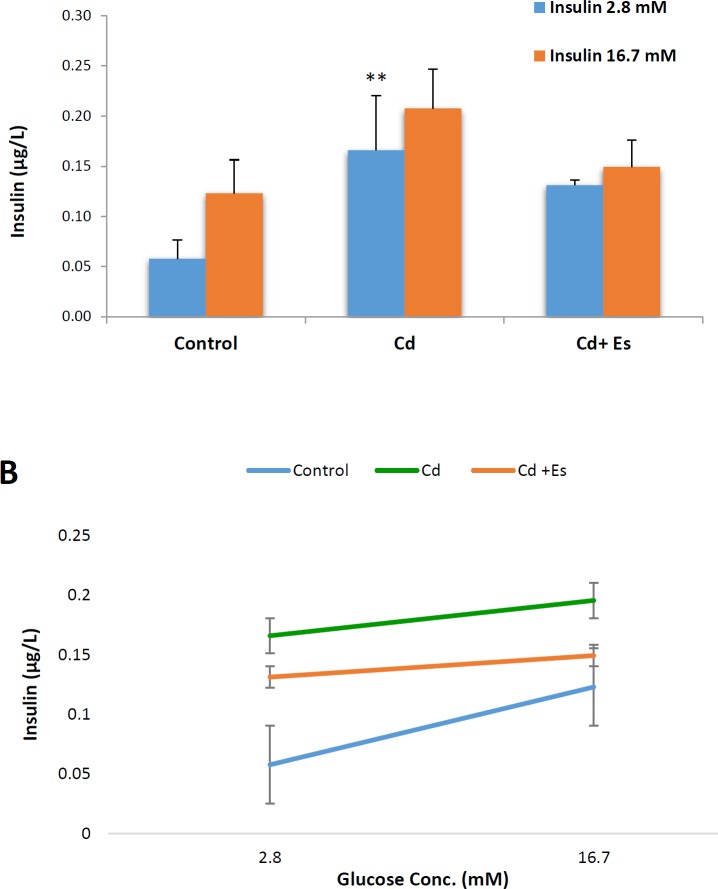
Effects of cadmium chloride and β-estradiol on the release of insulin from the islets of Langerhans isolated from the pancreas after 24 hr of exposure. Isolated islets were incubated for 1 hr with basal concentration of glucose (2.8 mM) and stimulant concentration (16.7mM). Data are expressed as mean±SEM with a replication number, n= 3. ** Significantly different from control at *P*-value < 0.01. Cd: cadmium, Es: β-estradiol

**Figure 5:**
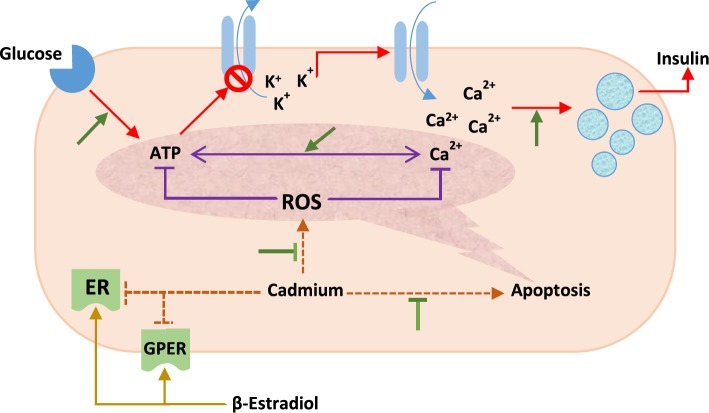
A schematic figure showing the interaction between cadmium and β-estradiol regarding their effects on cellular homeostasis and insulin secretion in the pancreatic β-cells. Red arrows indicate the process of glucose-stimulated insulin secretion. Green arrows indicate effects of estrogen receptor signaling cascade. ER: estrogen receptor; GPER: G-protein coupled estrogen receptor; ROS: reactive oxygen species

**Table 1:** Flowcytometry evaluation of rat pancreatic islets exposed to cadmium chloride and β-estradiol for the percentage of live, apoptotic, and necrotic cells

**Percentage** ** of cells (%)**	**Control**	**Cadmium**	**Cadmium + β-estradiol**
Live cells	82.02	50.92	58.72
Apoptotic cells	6.86	9.58	5.1
Necrotic cells	11.12	39.48	36.26

***Animal treatment***

All experiments involving laboratory animals matched the guidelines of an institutional board. Wistar rats weighing 200–250 g (gender: male, age 2–3 months) were adapted to laboratory conditions one week before doing experiments. The animals were housed in the laboratory at a 12-hr dark/light cycle, 50% humidity, and 25 ± 1 ^°^C.

***Isolating islets of Langerhans from the pancreas***

Before starting any of the experiments, the animals were allowed to spend an acceptable period of time in the lab environment. For induction of anesthesia, the rats were intraperitoneally injected a combination of ketamine and xylazine in a ratio of 10:1 based on the doses calculated for ketamine (100 mg/kg) and xylazine (10 mg/kg). After complete anesthetization, a surgical incision was performed in the abdominal cavity of the animals and the pancreatic duct was channelized to inject a cold and fresh Krebs buffer. Perfusion of the pancreas with Krebs buffer made it baggy and easy to isolate. When the pancreas and its connection was isolated, it was chopped into small pieces in the cool Krebs buffer and centrifuged for 60 sec at 3000g and 4 ^°^C. Then, the sediment was mixed with the collagenase enzyme and shaken for 10 min in a 37 ^°^C water bath for complete separation of islets. After stopping the digestion with BSA, the sediments were washed twice with Krebs buffer and islets of Langerhans were observed and picked up under a stereomicroscope. Finally, the healthy isolated islets were cultured in the RPMI 1640 medium at 37 ^°^C for 24 hr in order to overcome side effects from the stressful procedure of isolation (10). 

***Treatment of islets***

Langerhans islets were divided into groups of 10-digit and each group was treated with a special concentration of the materials for 24 hr at 37 ^°^C. In order to find median lethal concentration (LC_50_) of cadmium chloride, seven concentrations of cadmium chloride in a logarithmic scale ranging from 1 nM to 1 mM were prepared in the RPMI medium and each group of islets was treated with a special concentration of cadmium chloride. Such a dose-response experiment was done using five concentrations of β-estradiol ranging from 0.01 nM to 100 nM in order to find the effective concentration of β-estradiol in the islets exposed to LC_50_ of cadmium chloride. In the following experiments, islets were grouped as control, cadmium chloride, and cadmium chloride plus β-estradiol, and biomarkers of oxidative stress, apoptosis; secretory function of β-cells was evaluated in this experimental setting. 

***Cell viability assessment***

MTT, a yellow tetrazole, can be reduced to purple formazan, by NAD(P)H-dependent cellular oxidoreductase enzymes which reflect the metabolic activity of viable cells. When the treatment period was completed, the medium was removed and the islets were washed with Krebs/HEPES buffer two times. Then 50 μl of MTT (0.5 mg/ml) was added to each group and the islets were incubated at 37 ^°^C for 4 hr. DMSO was added and the islets were shaken for 30 min at room temperature. Finally, the absorbance of the samples was measured at the wavelength of 570 nm by a microplate reader (11).

***Flow cytometry assessment of apoptosis vs necrosis***

When the treatment period was completed, islets of Langerhans were transformed into single cells by using trypsin. In the following, blockade of trypsin-induced digestion was performed by adding BSA and washing cell suspension with phosphate buffered saline (PBS) two times. Dual staining with the ApoFlowEx® FITC kit was used to assess apoptosis vs necrosis. 5 μl of annexin V–fluorescein isothiocyanate (FITC) and 5 μl of propidium iodide (PI) were added to the cell suspensions at the concentration of 3 × 10^5^ cells/100 μl and incubation was done at room temperature for 15 min. Finally, the samples were injected into the flowcytometer (Apogee Flow Systems, UK) and the percentages of live, early apoptotic, late apoptotic, and necrotic cells were analyzed differentially (12).

***Measurement of cytosolic reactive oxygen species (ROS)***

Dichlorodifluorcein diacetate (DCFH-DA) is a non-fluorescent chemical that is deacetylated and oxidized to a fluorescent compound by ROS. After completing the treatment, the groups of islets were homogenized with an extraction buffer and centrifuged at 2375 for 15 min. 50 microliters of the supernatant was removed and added to the 162 microliters of an assay buffer. After adding 10 microliters DCFH-DA, incubation of the solution was done for 15 min at 37 ^°^C. Eventually, a microplate reader equipped to the fluorescence analyzer was used to measure the absorbance of the solutions every 10 min up to 60 min. Protein concentration was measured using the remaining islets in this protocol. The concentration of proteins was used to normalize the level of the other biomarkers as described previously (13).

***Assessment of insulin secretion***

When completing treatment, the groups of islets were translocated to the vials containing 1 ml Krebs medium. The vials were centrifuged at 3000g for 1 min and then the supernatants were removed and disposed of. The remaining islets were incubated with a Krebs medium containing 2.8 mM glucose for 30 min. Then, the contents of the vials were divided into two groups: 1. Basal insulin secretion assay and 2. Stimulated insulin secretion assay. The first groups received glucose at 2.8 mM concentration while the second groups of islets received glucose at 16.7 mM concentration. After 1 hr incubation and centrifugation of the vials, the supernatants were used to assess the concentration of insulin by using an insulin ELISA kit based on the manufacturer’s guidelines (14).

***Protein concentration assay***

Total protein concentration was measured to normalize the enzymatic activities and ROS levels. BSA was used as a standard protein, which was concentrated between 0 and 10 μg/ml in a buffer. Furthermore, samples were prepared by diluting 5 μl of supernatant with 795 μl distilled water. Then 200 μl of Bradford reagent was added to the samples and standards. After 5 min incubation, the absorbance of the solutions was measured at the 595 nm wavelength using a microplate reader (12).

***Statistical analysis of data***

The results were reported as the values of the mean ± standard error of three experiments in each group. The comparison between groups was done by one-way analysis of variance (ANOVA). In the following, the Bonferroni t-test (*post hoc*) was used to do multiple comparisons and determine *P*-values. Significant differences between treatment and control groups in each experiment were reported at three statistics of *P*-values (< 0.05, < 0.01, and < 0.001). 

## Results

***Viability of the islets of Langerhans***

First of all, dose-response assessments were performed in order to obtain the median lethal concentration of cadmium chloride and the protective concentration of β-estradiol on the islets of Langerhans in this experimental setup. Isolated islets of Langerhans from the pancreas of rats were treated with cadmium chloride at the logarithmic scale of concentrations ranging from 1 nM to 1 mM for 24 hr and were tested using MTT dye to evaluate the viability of the islets. As shown in Figure 1A, cadmium chloride at concentration 1 nM slightly decreased cellular viability of islets, so that 100 nM cadmium chloride caused around 50 % decrease in the cellular viability. In this way, the concentration 100 nM was established as the median lethal concentration (LC_50_) of the cadmium chloride for islets of Langerhans isolated from the rat pancreas in this experimental set up (Figure 1A).

In order to obtain the effective concentration of β-estradiol against cadmium chloride, physiologic and pharmacologic concentrations of β-estradiol ranging from 0.01 nM to 100 nM in a logarithmic scale were tested against LC_50_ of cadmium chloride. Alterations in the viability of islets treated with cadmium chloride and β-estradiol have been presented in Figure 1B. As shown, β-estradiol at pharmacologic concentrations including 1 nM and more was unable to raise the decreased viability of the islets, while physiologic concentrations of β-estradiol, which are less than 1 nM (0.01 and 0.1 nM) especially 0.01 nM strongly elevated cellular viability of pancreatic islets treated with cadmium chloride (Figure 1B). 

After performing dose-response assessments, established concentrations of chemicals including 100 nM cadmium chloride and 0.01 nM β-estradiol were used in the following experiments. 

***ROS formation in the islets of Langerhans***

Triplet groups of pancreatic islets were exposed to cadmium chloride and cadmium chloride plus β-estradiol, and after 24 hr incubation, the cellular level of reactive oxygen species (ROS) was measured with the fluorescent dye dichlorodifluorcein diacetate (DCFH-DA). In fact, DCFH-DA is a fluorogenic compound that is used to measure the activities of ROS such as hydroxyl and peroxyl radicals within the cells. When diffused into the cells, DCFH-DA is de-acetylated by esterase enzymes to DCFH, which is yet non-fluorescent. ROS can oxidize DCFH into a potent fluorescent compound, DCF, which can be detected by fluorescence spectrometry with maximum excitation of 495 nm and maximum emission of 529 nm in the spectra. As shown in Figure 2, cadmium chloride increased the amount of ROS in the islets while milder elevation in the ROS level happened when the islets were co-treated with β-estradiol and cadmium chloride. This result implicates the oxidative effect of cadmium chloride through increasing ROS production and meanwhile, ameliorative effects of β-estradiol on cadmium-induced oxidative stress (Figure 2). 

***Apoptosis vs necrosis in the islets of Langerhans***

In order to evaluate the rate of apoptosis and necrosis during the process of islet exposure to cadmium chloride and β-estradiol, we performed a flowcytometric assay using annexin V–FITC. Actually, annexin V–FITC can conjugate with phosphatidylserine transferred from the inner part of the cell membrane to the outer part of the cell membrane in the phase of apoptosis. As plotted in Figure 3A, the lower left field shows the percentage of live cells, while the lower right and the upper right fields are representative of early apoptosis and late apoptosis, respectively. The upper left field also shows the percentage of necrotic cells in each group (Figure 3A). As quantified in Figure 3B, the percentage of live cells has been decreased from 82% to 50% under the effect of cadmium chloride, but this decrease in the number of live cells was modified up to 58% when cadmium chloride was co-administered with β-estradiol to the islets. In the control group of islets, total percentage of apoptotic cells was 6.86%, while in the other two groups including cadmium and cadmium plus β-estradiol-treated islets apoptosis was measured as 9.58% and 5.01%, respectively. With regard to the reduced percentage of apoptotic cells under the effect of β-estradiol in comparison with the cadmium treated group and even the control group, it seems that β-estradiol has anti-apoptotic effects in the pancreatic islets. Regarding necrotic cells, there is also an increasing trend from 11.12% up to 39.48% under the effect of cadmium chloride. This increasing trend has been modified by β-estradiol to 36.26%, which is representative of a slight protective effect of β-estradiol against necrosis of pancreatic islets exposed to cadmium chloride (Table 1).

***Insulin secretion from the islets of Langerhans***

The effects of cadmium chloride and β-estradiol on insulin secretion from pancreatic islets were evaluated at both basal and stimulated phases and the results have been presented in Figure 4. Generally, insulin secretion was increased under the effect of our experimental chemicals, but this increment was sharper when cadmium chloride was used to treat islets alone than when used along with β-estradiol. It seems β-estradiol relieves somewhat intensifying effect of cadmium chloride on insulin secretion from the pancreatic islets particularly at the stimulated phase (Figure 4). 

## Discussion

The heavy metal cadmium is one of the known environmental pollutants that can cause many health problems for humans. Significant epidemiological and experimental evidence suggests that exposure to cadmium can increase the risk of diabetes in humans but the exact toxicological mechanism especially pancreatic β-cell damage remains unexplained. 

The objective of this work was to evaluate the gluco-toxic mechanism of cadmium according to the interaction of this metallo-hormone with estrogen receptors from one side, and presence and biological functions of estrogen receptors in the pancreatic β-cells on the other side. 

In the previous works on the rodent models of both type 1 diabetes and type 2 diabetes, β-estradiol has been shown to protect pancreatic β-cells against oxidative elements and apoptosis, as such findings have inspired researchers to consider pancreatic estrogen receptors as a promising approach for the treatment of diabetes in the future (8). In this way, β-estradiol, as an endogenous agonist of estrogen receptors, was used along with cadmium chloride to treat islets of Langerhans isolated from rats, and the results showed that activating estrogen receptors in the pancreas alleviated toxic effects of cadmium. 

MTT assay was used to evaluate the viability of islets and to conduct dose-response experiments in order to find the toxic concentration (LC_50_) of cadmium and effective concentration of β-estradiol. 100 nM was established as the LC_50_ of cadmium chloride, while the effective concentration of β-estradiol against cellular toxicity of cadmium chloride was found in the range of its physiologic concentrations especially 0.01 nM so that elevated levels up to the pharmacologic concentrations decreased the efficacy of β-estradiol. Protective effect of β-estradiol on the cellular viability of pancreatic islets at physiological, not pharmacological concentrations, can be an explanation regarding the lower prevalence of diabetes in premenopausal women than in men (15). This effect of β-estradiol confirms the result of the previous works implicating the protective role of estrogen receptors in the integrity and physiological functions of the endocrine pancreas. 

Our finding regarding the formation of ROS from the islets of Langerhans was also in parallel with the results of cellular viability assay indicating the pro-oxidative effect of cadmium chloride and antioxidant effect of β-estradiol in the islets. Cadmium chloride is known to manifest its cellular toxicity by disturbing intracellular redox status and induction of oxidative stress as the same result was achieved in this experiment on ROS production in the pancreatic islets. Increasing effect of cadmium chloride on the production of ROS in the islets of Langerhans was prevented to some extent by β-estradiol implicating the role of estrogen receptors in the protective effect of β-estradiol against oxidative stress. It has already been reported that estradiol mediates its antioxidant effects through activating estrogen receptors alpha and beta and up-regulation of protein and activity of superoxide dismutase and catalase (16, 17). 

The results of the current work taken from flowcytometric assay also revealed respective pro- and anti-apoptotic effects for cadmium chloride and β-estradiol against each other in the islets of Langerhans. Cadmium chloride-induced cell necrosis has also been shown to decrease by β-estradiol, but alleviating the effect of β-estradiol against cadmium-induced apoptosis especially in the late phase was more remarkable.

In addition to the cellular viability, function of islets of Langerhans focusing on β-cells was tested by measurement of insulin secretion into the media under the basal and glucose-stimulated conditions. Insulin, as the exclusive blood glucose concentration lowering hormone, has a regulated secretion under the effect of blood glucose concentration and some other stimuli. In fact, pancreatic β-cells detect extracellular glucose concentration and release insulin as required. Upon transporting glucose into the β-cells and elevation of cellular ATP/ADP ratio, ATP dependent potassium channels are closed and action potential occurs in the cell membrane. Depolarization of cell membrane causes calcium channels to open, and the influx of calcium ions into the cells leads to vesicular release of insulin into the blood. For this reason, the amount of insulin secreted into the blood under the postprandial glucose concentration is far more than that of fasting blood glucose. That is why insulin secretion has a biphasic model and is investigated at both basal and glucose-stimulated concentrations. The results (Figure 4) showed that both basal and glucose-stimulated insulin secretions have been increased by cadmium and cadmium plus β-estradiol, although the increasing effect of cadmium on insulin secretion was alleviated by β-estradiol. One explanation for the increasing effect of cadmium on insulin secretion can be due to elevated production of ROS in the islets of Langerhans treated with cadmium. It has been reported that ROS can stimulate intracellular ryanodine receptors and causes the release of calcium ions leading to release of insulin (18). 

On the other side, increasing effect of cadmium on insulin content of the media has occurred at the concentration at which the cells experienced a 50% decrease in the viability due to apoptosis or necrosis. An elevated level of insulin following exposure to a toxic concentration of cadmium can be considered a compensatory response against cellular damage to the endocrine pancreas that tries to signal and repair the injury by secreting insulin as a growth factor. 

Moreover, it may be proposed that increasing concentration of insulin in the media may be due to releasing hormonal content of the pancreatic β-cells after decomposition of pancreatic islets. 

However, decreasing effect of β-estradiol against cadmium-induced insulin release can be mediated by preserving the viability and integrity of pancreatic β-cells, as well as decreased production of ROS. Furthermore, trends of insulin secretion after stimulating pancreatic islets with high concentration of glucose (16.7 mM) have been plotted in Figure 4B, and comparison of the insulin levels of these three groups in basal and glucose-stimulated states, reveals that pancreatic islets treated with cadmium chloride have lower ability to increase insulin secretion after stimulation with high concentration glucose. Decreased line slope related to the cadmium treated group in comparison with that of the control group is representative of this point (Figure 4B). The line slope for that group of islets treated with cadmium chloride plus β-estradiol has been increased when compared with cadmium treated group, though this increment is not significant. However, this decrease in the ability of islets in secreting insulin after stimulating with glucose can be considered as a reduced sensitivity of pancreatic β-cells, which can be due to disturbance in the glucose signaling cascade induced by cadmium (Figure 5).

## Conclusion

It can be expected that modification in the viability, integrity, and function of pancreatic islets and in particular β-cells is determinant in finding new mechanistic approaches for cellular targets influenced by both developing and preventing factors in diabetes. The results of this work focusing on the viability and functionality of the islets under the effect of β-estradiol against toxicity of a metallo-hormone, cadmium, offers pancreatic estrogen receptors as a target that can be modified by both toxic and protective agents and interaction with these receptors is worth searching and exploring in the pharmacology and toxicology of diabetes. 
